# Altered IL-3 and lipocalin-2 levels are associated with the pathophysiology of major depressive disorder: a case-control study

**DOI:** 10.1186/s12888-023-05354-y

**Published:** 2023-11-13

**Authors:** Mst. Sarmin Akter, Faisal Abdullah Emon, Zabun Nahar, MMA Shalahuddin Qusar, Sardar Mohammad Ashraful Islam, Mohammad Shahriar, Mohiuddin Ahmed Bhuiyan, Md. Rabiul Islam

**Affiliations:** 1 Department of Pharmacy, University of Asia Pacific, 74/A Green Road, Farmgate, Dhaka, 1205 Bangladesh; 2 Department of Psychiatry, Bangabandhu Sheikh Mujib Medical University, Shahabagh, Dhaka, 1000 Bangladesh; 3 School of Pharmacy, BRAC University, 66 Mohakhali, Dhaka, 1212 Bangladesh

**Keywords:** Major depressive disorder, Interleukin-3, Lipocalin, Cytokines, Case-control study

## Abstract

**Background:**

Major Depressive Disorder (MDD) is a common mental ailment and is the primary reason for disability. It manifests a severe impact on moods, thoughts, and physical health. At present, this disorder has become a concern in the field of public health. Alteration of neurochemicals is thought to be involved in the pathogenesis of many psychiatric disorders. Therefore, we aimed to evaluate serum IL-3 and lipocalin-2 in MDD patients and healthy controls (HCs).

**Method:**

We included a total of 376 participants in this study. Among them, 196 were MDD patients, and 180 were age-sex-matched HCs. MDD patients were recruited from the Psychiatry Department of Bangabandhu Sheikh Mujib Medical University (BSMMU), but the controls were from different parts of Dhaka. All study participants were evaluated by a psychiatrist using the DSM-5 criteria. To assess the severity of the depression, we used the Hamilton depression (Ham-D) rating scale. Serum IL-3 and lipocalin-2 levels were measured using commercially available enzyme-linked immune-sorbent assay kits (ELISA kits).

**Results:**

According to this study, we observed elevated serum levels of IL-3 (1,024.73 ± 29.84 pg/mL) and reduced levels of serum lipocalin-2 (29.019 ± 2.073 ng/mL) in MDD patients compared to HCs (911.11 ± 20.55 pg/mL and 48.065 ± 3.583 ng/mL, respectively). No associations between serum levels of IL-3 and lipocalin-2 and depression severity were observed in patients.

**Conclusions:**

According to the present findings, alterations of serum IL-3 and lipocalin might be associated with the pathogenesis of MDD. These results support that altered serum neurochemicals can serve as early risk assessment markers for depression. Further interventional studies are recommended for a better understanding of the role of IL-3 and lipocalin-2 in the pathophysiology of depression.

## Background

Major depressive disorder (MDD) is a well-known, complex mood disorder with high rates of morbidity, recurrence, disability, and suicide that causes changes in mood, thoughts, behavior, and physical health [1, 2]. It is a widespread and severe mental disorder that can affect a person to enjoy life and reduce the ability to perform daily duties. It is also associated with other physical and mental illnesses [3]. A study reported that 970 million people worldwide suffer from different mental disorders. Among them, 350 million suffer from MDD [4, 5]. The number of people living with depression worldwide increased by 50% from 1990 to 2017 [6]. MDD is expected to be the leading cause of the burden of all medical disorders across the world by 2030 [7]. Depression results from a complex interaction of genetic, social, psychological, biological, and environmental factors [8]. People who have gone through adverse life events (unemployment, financial insecurity, chronic or life-threatening health problems, exposure to violence, social separation, sadness, etc.) are more likely to develop depression [9]. The lifetime prevalence of depression is higher in women (20-25%) than in men (7-12%) [10]. Evidence suggests that patients with a family history of MDD in first-degree relatives have up to 3 times higher risk of developing the disease than those who do not have it [11]. MDD remains either undertreated or untreated even though it is a prevalent disease [12]. The primary healthcare provider is the first person responsible for the diagnosis of depression. However, evidence suggests that it remains unrecognized at this level due to poor understanding of the disease mechanism by primary healthcare professionals. Since the diagnosis of depression mostly depends on statements by patients, this leads to misdiagnosis and confusion [13]. To successfully treat depression, the first step is to recognize the problem and make a correct diagnosis [14].

Major depression is triggered by genetic, metabolic, endocrine, neurobiological, and environmental factors [15]. Several earlier studies reported that depressed patients might have increased levels of cytokines or other inflammatory parameters in blood and cerebrospinal fluid [16]. Though the etiology and pathophysiology of MDD remain unclear, numerous reports have suggested the association between neuroinflammation and depression [17, 18]. Inflammation is a complex biological response to infection, injury, and other organismal stress. It involves the activation process of immune systems that alter cytokine levels [15]. Cytokines and other inflammatory molecules can cross the blood-brain barrier either by crossing through weak pores of the blood-brain barrier or by binding to the transport molecules on it. The pro-inflammatory mediators in the brain can affect brain function by several mechanisms [19]. They can inhibit the release of neurotransmitters from the presynaptic neuron. These neurotransmitters include dopamine (DA), serotonin (5-hydroxytryptamine, 5HT), and norepinephrine (NE) play a fundamental role in mood and anxiety disorders [20]. People experience pleasure mediated by the neurotransmitter dopamine. Many studies reported the decreased levels of DA, 5HT, and NE metabolites in serum and cerebrospinal fluid of depressed patients [21–23]. The increased level of inflammatory cytokine alters the metabolism of dopamine, serotonin, and noradrenaline which activate the hypothalamic-pituitary-adrenal (HPA) axis to cause depression [15]. The HPA axis is a neuroendocrine stress response system for maintaining stability and health [24]. In contrast to pro-inflammatory cytokines, the levels of anti-inflammatory cytokines (IL-4, IL-10, IL-13, etc.) are reduced in MDD patients [25]. Several studies reported that the altered levels of some pro-inflammatory and anti-inflammatory cytokines are responsible for the pathogenesis of depression [15]. However, the knowledge about biological markers for understanding the pathophysiology of MDD is limited [26].

Altered levels of cytokines, for example, IL-3, lipocalin-2, tumor necrosis factor-alpha (TNF-α), IL-1β, and IL-6, have been reported in patients with MDD [17]. IL-3 is a cytokine with multiple biological functions in immune response, including in the suppression of apoptosis, stimulation of cell growth, and development and differentiation of all hematopoietic cell types [27]. IL-3 may affect inflammatory responses in the developing or mature brain [28]. Abnormal serum levels of IL-3 have been observed in patients with mood disorders [29, 30]. Furthermore, according to previous studies, IL-3 and IL-3R are expressed in the brain cells of patients with mood disorders [31, 32]. Lipocalin-2 is known as neutrophil gelatinase-associated lipocalin (NGAL) which has recently been identified as a newly discovered adipokine (cytokine) that is highly expressed in the white adipose tissue [33]. Lipocalin-2 has association with neuroinflammation. Therefore, it might play a role in developing depression, mild cognitive impairment, and neurological disorders [34, 35]. Interestingly, the induction of a peripheral inflammatory response and psychological stressors cause cerebral expression of lipocalin-2, which may consequently reduce spinal synaptic density in the hippocampus. Together, these data suggest that increased levels of lipocalin-2 in the central nervous system may lead to decreased neuroplasticity [33, 34, 36]. Altered cytokines may lead to synaptic degeneration and neuronal cell death. The association between cytokine dysregulation and neurological disorders is evident [37]. Therefore, altered serum levels of IL-3 have been observed in patients with mood disorders [29, 30] and lipocalin-2 in patients with late-life depression [33, 34, 36]. Thus, serum IL-3 and lipocalin-2 levels can serve as tools for diagnosis and early risk assessment of MDD. However, there are limited studies on the effects of IL-3 and lipocalin-2 in MDD patients. The present study aimed to evaluate serum IL-3 and lipocalin-2 in MDD patients and healthy controls (HCs) to understand their role in the pathophysiology and development of depression.

## Methods

### Study design and participants

This prospective case-control study enrolled 196 MDD patients and 180 HCs matched by age and sex. We recruited MDD patients between 18 and 60 years suffering from depressive symptoms for at least two weeks or longer in this study from the Department of Psychiatry, Bangabandhu Sheikh Mujib Medical University (BSMMU), Dhaka, Bangladesh. BSMMU is a tertiary-level health care center in the capital city that provides general and specialized treatments to mass people. Patients of every age, sex, educational status, and financial condition from all over Bangladesh come here to seek medical aid. HCs were from different parts of Dhaka city. We included the study subjects through randomization. The study participants were evaluated by a qualified psychiatrist according to the Diagnostic and Statistical Manual of Mental Disorders, 5th Edition (DSM-5). A predesigned structured questionnaire was used to record the socio-demographic and biographical profiles of subjects. The severity of depression was measured by the Hamilton depression (Ham-D) rating scale. A Ham-D score of 7 or greater was considered as case. Subjects with comorbid psychiatric illness or other neuropsychiatric disorders, abnormal body mass index (BMI), immune disorders, infectious diseases, and alcohol or drug dependency were excluded from this study. We also excluded subjects currently on psychiatric medications. Exclusion criteria were equally applied to both cases and controls.

### Blood sample collection

Following an overnight fast, 5 ml of blood sample was drawn from the cephalic vein of each subject by venipuncture between 8:00 AM and 9.00 AM and collected into falcon tubes. After collection, the blood samples were kept in a fixed place at room temperature for one hour without shaking or agitation and allowed to clot. Then clotted blood samples were centrifuged at 1000×g for 15 min, and serum samples were collected into an Eppendorf tube and stored at − 80 ^0^ C until further analysis.

### Serum analysis

Serum IL-3 and lipocalin-2 levels were measured using commercially available human enzyme-linked immunosorbent assay (ELISA) kits (BosterBio, USA) following the instructions provided by the manufacturer. The sensitivity or minimum detectable range of IL-3 and lipocalin-2 were < 1 pg/ml and < 10 pg/ml, respectively. There was no cross-reactivity with the other cytokines present in the serum. Analysis of all samples was performed by a person who was blind to the clinical status of the participants.

### Statistical analysis

All data were processed by Microsoft Excel and SPSS software package version 25.0 (IBM Corporation, Armonk, USA) was used to conduct all statistical analyses. An Independent sample t-test was applied for numerical variables to see the group differences. Whereas the chi-square test was applied for categorical variables. Also, Spearman’s correlation test was used to determine the relationship among the variables. Bonferroni-corrected p-values were calculated for pairwise comparisons in correlation analysis. We constructed scatterplot and boxplot graphs to see the distribution pattern of the analyzed parameters. All analyses were considered significant if the p-value was less than or equal to 0.05.

## Results

### Socio-demographic characteristics of study population

The socio-demographic profiles of the study population are summarized in Table 1. There was no significant difference between MDD patients and HCs regarding age (MDD patients: 30.86 ± 0.69 years; HCs: 30.83 ± 0.70 years). Both study groups had a higher number of participants from the 18–25 and 23–35 age groups, where MDD patient counts were 36.22% and 37.76%, respectively, and healthy control counts were 35.00% and 36.67%, respectively, in the stated age ranges. The proportion of male (MDD patients: 33.16%; HCs: 29.44%) and female participants (MDD patients: 66.84%; HCs: 70.56%) was similar in both groups. We found that two-thirds of the MDD patients were married (66.33%), whereas the ratio is slightly less than half in HCs (47.22%). BMI was normal in the majority of the study subjects (MDD patients: 53.57%; HCs: 56.11%). In terms of education, the maximum number of participants in the MDD groups had a secondary education level (38.27%), whereas HCs had a graduation or higher degree (43.89%). Higher numbers of study subjects in MDD groups were unemployed (27.55%), but HCs subjects were commonly students (31.11%) among other occupational groups. Most study subjects, both MDD patients and HCs, had a medium economic impression (MDD patients: 72.96%; HCs: 53.33%). A superior number of the study subjects were from urban areas (MDD patients: 57.14%; HCs: 76.11%) and primarily nonsmokers (MDD patients: 90.82%; HCs: 91.11%). Despite having a lower family history of MDD (27.04%), most MDD patients were previously diagnosed with MDD (55.61%). None of the study subjects from HCs had a previous history of MDD, and very few had a family history of MDD.

**Table 1:** Socio-demographic profile of the study population

Characteristics	MDD patients (n = 196) Mean ± SEM	Healthy controls (n = 180) Mean ± SEM	*P value*
Age in years	30.86 ± 0.69	30.82 ± 0.70	0.963
18–25	71 (36.22%)	63 (35.00%)	
26–35	74 (37.76%)	66 (36.67%)	
36–45	33 (16.84%)	36 (20.00%)	
46–60	18 (9.18%)	15 (8.33%)	
Sex			0.653
Male	65 (33.16%)	53 (29.44%)	
Female	131 (66.84%)	127 (70.56%)	
Marital Status			< 0.001
Married	130 (66.33%)	85 (47.22%)	
Unmarried	66 (33.67%)	95 (52.78%)	
BMI (kg/m^2^)	23.64 ± 0.32	24.31 ± 0.28	0.123
Below 18.5 (CED)	21 (10.71%)	7 (3.89%)	
18.5–25 (normal)	105 (53.57%)	101 (56.11%)	
Above 25 (obese)	70 (35.72%)	72 (40.00%)	
Education level			0.003
Illiterate	15 (7.65%)	14 (7.78%)	
Primary level	50 (25.51%)	23 (12.78%)	
Secondary level	75 (38.27%)	64 (35.55%)	
Graduate and above	56 (28.57%)	79 (43.89%)	
Occupation			< 0.001
Business	7 (3.57%)	4 (2.22%)	
Service	30 (15.31%)	47 (26.11%)	
Housewife	46 (23.47%)	23 (12.78%)	
Unemployed	54 (27.55%)	35 (19.45%)	
Student	24 (12.24%)	56 (31.11%)	
Others	35 (17.86%)	15 (8.33%)	
Economic impression			< 0.001
High	21 (10.71%)	78 (43.33%)	
Medium	143 (72.96%)	96 (53.33%)	
Low	32 (16.33%)	6 (3.34%)	
Smoking habit			0.921
Smoker	18 (19.18%)	16 (8.89%)	
Non smoker	178 (90.82%)	164 (91.11%)	
Residence area			< 0.001
Rural	84 (42.86%)	43 (23.89%)	
Urban	112 (57.14%)	137 (76.11%)	
Previous history of MDD			< 0.001
Yes	109 (55.61%)	0 (0.00%)	
No	87 (44.39%)	180 (100.00%)	
Family history of MDD			< 0.001
Yes	53 (27.04%)	2 (1.11%)	
No	143 (72.96%)	178 (98.89%)	

Abbreviations: BMI, body mass index; CED, chronic energy deficiency; MDD, major depressive disorder; SEM, standard error mean

### Laboratory findings of the study

**Fig. 1:**
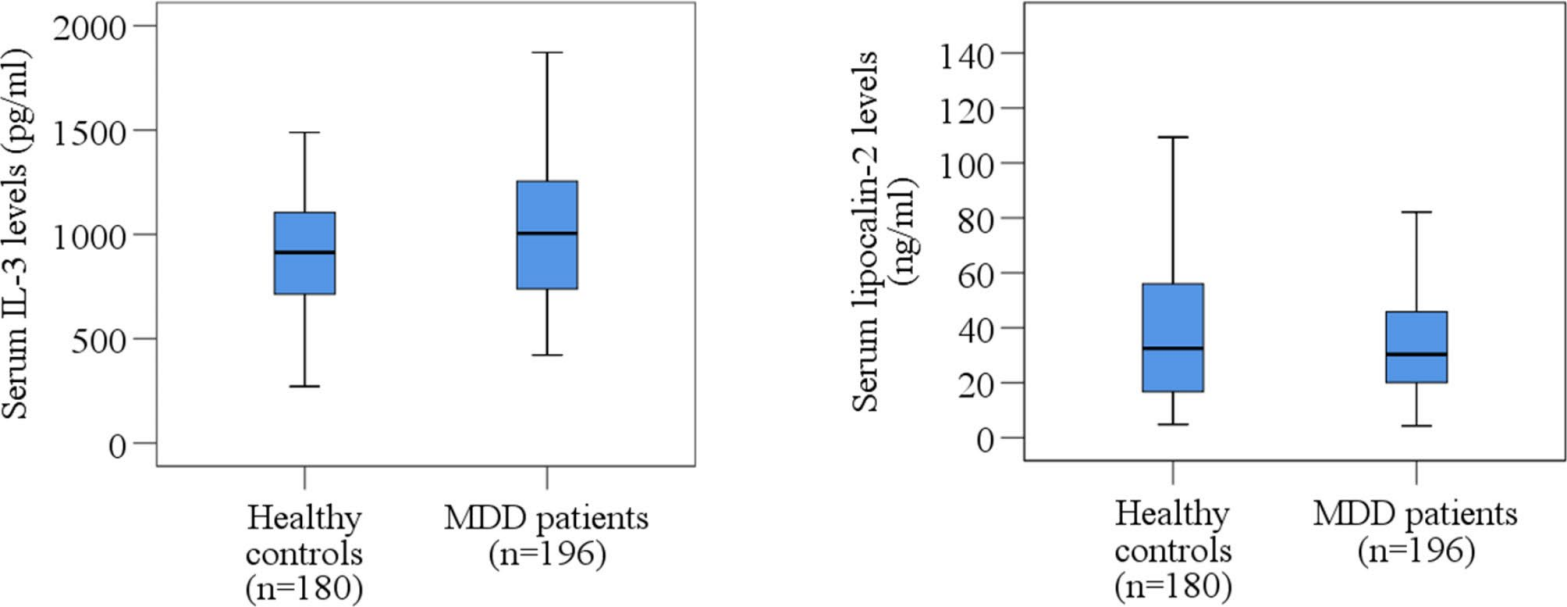
Distribution of serum IL-3 and lipocalin-2 levels in MDD patients and healthy controls. Boxplot graphs showing the median, maximum and minimum value range

**Fig. 2:**
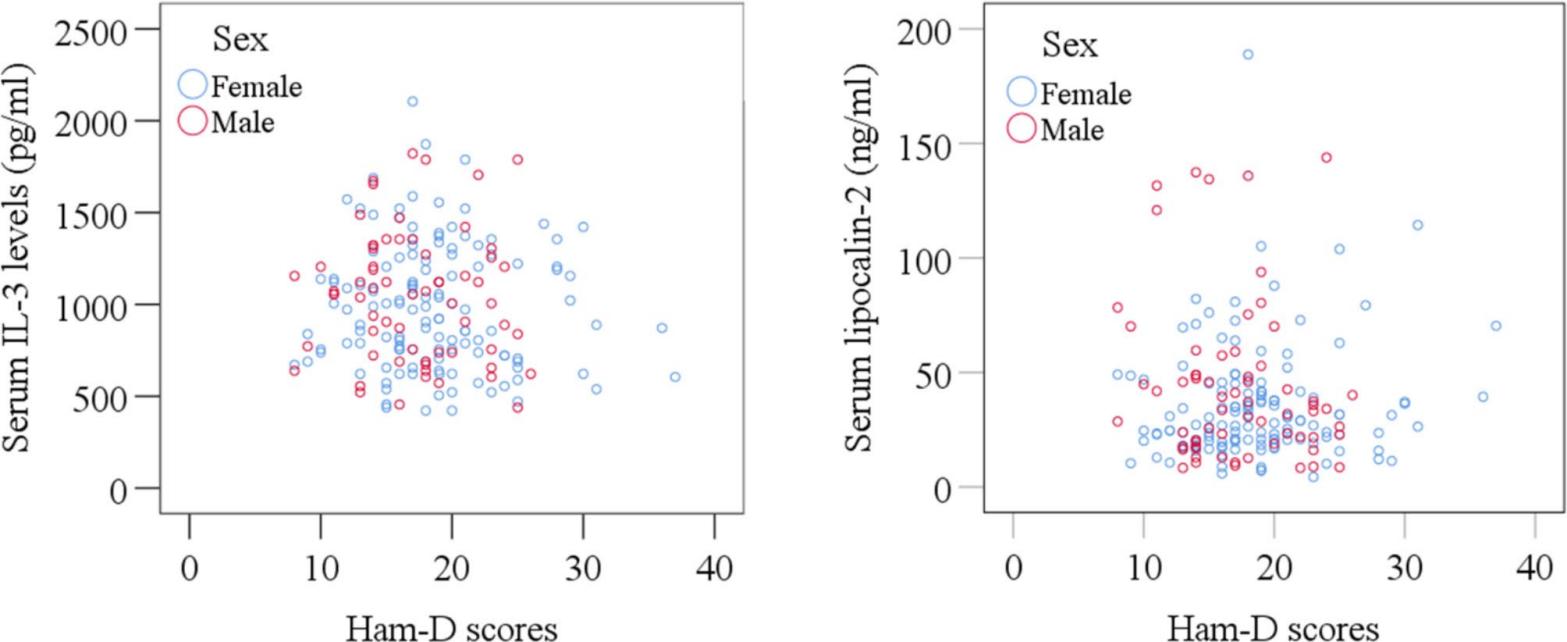
Sex-specific scatter plot graphs showing the distribution of serum IL-3 and lipocalin-2 levels with Ham-D scores of MDD patients

The clinical outcomes and laboratory findings of this study are shown in Table 2. We found higher serum IL-3 levels in MDD patients than HCs (1,024.73 ± 29.84 pg/mL vs. 911.11 ± 20.553; p = 0.002). Additionally, we observed serum IL-3 levels of females were significantly higher in MDD patients than in HCs. However, the MDD patients had lower mean serum lipocalin-2 levels than HCs (38.03 ± 2.07 ng/mL vs. 47.10 ± 3.58 ng/mL; p = 0.026). Also, serum lipocalin-2 levels of females were significantly lower in MDD patients than in HCs. Figure 1 shows the alteration of serum IL-3 and lipocalin-2 levels. Patients with MDD display greater mean IL-3 concentrations than HCs in both sexes. Alterations in serum IL-3 and lipocalin-2 were not linked to severity scores (Ham-D score) in MDD patients. According to Spearman’s correlation, we observed only serum IL-3 levels were positively associated with BMI among all pairwise comparisons after Bonferroni correction of p values (r = 0.216; p = 0.018) (Table 3). Female MDD patients had higher serum IL-3 and lower serum lipocalin-2 levels at higher Ham-D scores, but regarding lipocalin-2, male MDD patients had the opposite pattern, according to sex-specific scatter plot graphs (Fig. 2).

**Table 2:** Clinical profile and laboratory findings of the study population

Parameters	MDD patients (n = 196) Mean ± SEM	Healthy controls (n = 180) Mean ± SEM	*p value*
Age (years)	30.86 ± 0.69	30.82 ± 0.70	0.963
Male (P/C:65/53)	29.95 ± 1.23	31.04 ± 1.32	0.550
Female (P/C:131/127)	31.31 ± 0.83	30.72 ± 0.83	0.617
BMI (Kg/m2)	23.64 ± 0.32	24.31 ± 0.28	0.123
Male (P/C: 65/53)	23.42 ± 0.55	24.42 ± 0.53	0.198
Female (P/C:131/127)	23.75 ± 0.39	24.26 ± 0.37	0.330
Ham-D score	18.17 ± 0.37	2.26 ± 0.22	< 0.001
Male (P/C: 65/53)	17.17 ± 0.56	2.60 ± 0.48	< 0.001
Female (P/C:131/127)	18.67 ± 0.47	2.12 ± 0.25	< 0.001
Serum IL-3 (pg/mL)	1,024.73 ± 29.84	911.11 ± 20.55	0.002
Male (P/C: 65/53)	1,043.97 ± 43.51	944.31 ± 37.79	0.094
Female (P/C:131/127)	1,015.18 ± 39.16	897.26 ± 24.48	0.012
Serum lipocalin-2 (ng/mL)	38.03 ± 2.07	47.10 ± 3.58	0.026
Male (P/C: 65/53)	43.79 ± 4.35	40.66 ± 4.64	0.623
Female (P/C:131/127)	35.17 ± 2.2	49.79 ± 4.68	0.005

Abbreviations: BMI, body mass index; Ham-D, Hamilton depression rating scale; IL-3, interleukin-3; MDD, major depressive disorder; P/C, patients/control; SEM, standard error mean

**Table 3:** Spearman’s correlation study among various research parameters among MDD patients

*Correlation parameters*	*r*	p*
Age and Ham-D	-0.086	1.000
Age and IL-3	0.275	0.081
Age and lipocalin-2	-0.026	1.000
BMI and Ham-D	-0.047	1.000
BMI and IL-3	0.216	0.018
BMI and lipocalin-2	0.038	1.000
IL-3 and Ham-D	-0.087	1.000
Lipocalin-2 and Ham-D	-0.010	1.000
IL-3 and lipocalin-2	-0.162	0.207

BMI, body mass index; Ham-D, Hamilton depression rating scale; IL-3, interleukin-3; MDD, major depressive disorder. *Bonferroni-corrected p values

## Discussion

As far as we know, this is the first study on serum IL-3 and lipocalin-2 levels in MDD patients from Bangladesh. Identification and application of appropriate biomarkers are crucial for the successful diagnosis and treatment of MDD patients. Organized clinical interviewing is still the mainstay of diagnosing depression, as a reliable risk evaluation marker has yet to be identified [38, 39]. In this study, we investigated the potential value of IL-3 and lipocalin-2 levels as a marker for early risk assessment of MDD. Our findings indicate that serum IL-3 was significantly higher and lipocalin-2 levels were lower in MDD patients compared to HCs. Several biomarkers depict various components of the inflammatory system, which is incredibly complex. Numerous mechanisms, including stimulation of the hypothalamic-pituitary-adrenal axis (HPA axis) [40], cytokine receptor stimulation in neurons [41], activation of the kynurenine pathway [42], elevated serotonergic expression [43, 44], decreased neuronal growth factors, and changes in synthesis, release, and reuptake of neurotransmitter [45], could link cytokine-mediated immune activation to the pathophysiology of depression [46, 47]. Several studies on biomarkers, including pro- and anti-inflammatory cytokines, were conducted in previous decades to determine their association with depressive disorders [48]. These findings are supported by the connection between MDD and the neurological underpinnings of stress-induced alterations to the immune and nervous systems [49].

The Association between IL-3 and depression has not been investigated in Bangladeshi population yet but we found significantly higher IL-3 levels in the serum of MDD patients than HCs (p = 0.002) in our study. Some studies correlated elevated IL-3 levels in depressed patients of different populations [50]. Also, our findings support a previous study that found a positive correlation between high IL-3 levels and depressive symptoms in schizophrenia [51]. Antigen-activated T cells mostly produce IL-3, a cytokine that is the connection between immunity and hemopoietic system. It is related to various immune pathologies [52].

Additionally, other pro-inflammatory cytokines showed similar outcomes in depression like IL-1β, TNF- α, and IL-18 [53–55]. MDD patients had considerably higher levels of IL-1β and TNF-α than HCs, according to the findings of earlier studies [15, 56–59]. In addition, serum levels of IL-6, IL-10, and IL-18 were significantly higher in MDD patients [13, 60, 61]. Therefore, Increased IL-3 levels can contribute to the symptoms of neuropsychiatric illnesses like depression [51]. Moreover, our study observed that female MDD patients had serum IL-3 levels with significant elevation than the female controls (p = 0.012). Similar findings were observed in depressed females regarding other inflammatory cytokines like IL-17 A, IL-6, TNF-α [62]. The prevalence and severity of depression in women could be a contributing factor to our study findings.

Lipocalin-2 serves a variety of inflammation-related functions that are connected to autoimmune responses and persistent inflammation [63]. In this study, we found decreased lipocalin-2 levels in MDD patients. Also, the decrease in serum lipocalin-2 was significant in female MDD patients compared to female controls (p = 0.005). Although the mean lipocalin-2 level was found elevated in male patients than in male controls, that was not statistically significant. Men are reported to have higher plasma lipocalin-2 levels than women [64]. So, this gender-specific variability could be a reason for differences found in lipocalin-2 levels between males and females. Some studies showed that lipocalin-2 has anti-inflammatory activity [65] and play an antagonistic role in the effect of pro-inflammatory cytokines like IL-6 [66]. Also, a study revealed that depressive behaviors increase in case of a complete absence of lipocalin-2 in the body [67]. Moreover, the absence of lipocalin-2 makes the brain prone to inflammation [68]. So, here decreased expression of lipocalin-2 in the body may be associated with increased inflammatory activities leading to depression. Some earlier studies found the opposite result to our findings and suggested a correlation between elevated lipocalin-2 levels and late-life depression [33–36]. According to a different study, serum NGAL levels and depression indices are associated with persons with heart failure [33].

It has been demonstrated that high levels of cytokines cause symptoms similar to systemic depression, such as exhaustion, anorexia, weight loss, sleep difficulties, feelings of hopelessness, and decreased psychomotor activity [69, 70], and numerous studies have identified a relationship between various inflammatory and immunological biomarkers and depressive symptoms, or MDD [71]. Therefore, it is crucial to identify and define biological risk factors to understand the etiology and treatment of depression [72]. The finding of altered peripheral levels of IL-3 and lipocalin-2 in MDD patients may indicate a strong relationship between the pathophysiology of depression and these biomarkers. Additionally, analysis of serum IL-3 and lipocalin-2 levels provided significant diagnostic value for MDD patients and can be an effective indicator of probable depression development. The fact that age, sex, BMI, and other socio-demographic profiles have been firmly controlled between the groups is a positive aspect of the current study.

### Limitations

The current study has several drawbacks. We did not assess dietary habits, physical activity, and treatment outcomes on the analyzed parameters. However, the relatively small size of our study may generate results with statistical power. Here we measured serum IL-3 and lipocalin-2 levels at a one-time point in patients with MDD. Thus, we are uncertain whether the altered levels of IL-3 and lipocalin-2 are a state or trait marker. Moreover, evaluating serum IL-3 and lipocalin-2 levels does not reflect the entire neuroinflammatory process of MDD. Assessing other parameters within the same population and research facility setup would be suitable. Despite these limitations, our study has sufficient strengths. For instance, this is the first study conducted in Bangladesh to evaluate IL-3 and lipocalin-2. Only a few studies have been done about this pro-inflammatory cytokine globally, and some were with contradictory findings. This study ensured diversity and homogeneity among the study population.

## Conclusions

Altered serum IL-3 and lipocalin-2 levels might be associated with the pathophysiology of MDD. Therefore, these findings can pave a novel way to evaluate the risk of depression and potential treatment modalities. An observational study assessing peripheral markers may have a few limitations. Considering the results of the present study as preliminary, we recommend further research to examine the impact of the aforementioned markers on depression using larger, and more homogeneous samples.

## Data Availability

Data supporting our findings are available from the corresponding author on reasonable request.

## Abbreviations

5HT: 5-hydroxytryptamine
BMI: Body mass index
BSMMU: Bangabandhu Sheikh Mujib Medical University
DA: Dopamine
DSM-5: Diagnostic and statistical manual of mental disorders, 5th edition
ELISA: Enzyme-linked immunosorbent assay
Ham-D: Hamilton depression rating scale
HCs: Healthy controls
HPA: Hypothalamic-pituitary-adrenal
IL-3: Interleukin-3
MDD: Major depressive disorder
NE: Norepinephrine
SPSS: Statistical package for the social sciences

## References

[1] Nothdurfter C, Milenkovic VM, Sarubin N (2021). The cytokine IL-17A as a marker of treatment resistance in major depressive disorder?. Eur J Neurosci.

[2] Islam MR, Ali S, Karmoker JR (2020). Evaluation of serum amino acids and non-enzymatic antioxidants in drug-naïve first-episode major depressive disorder. BMC Psychiatry.

[3] Nasca C, Bigio B, Lee FS (2018). Acetyl-l-carnitine deficiency in patients with major depressive disorder. Proc Natl Acad Sci.

[4] Cheng P, Wang L, Xu L (2022). Factors related to the length of stay for major depressive disorder patients in China: a real-world retrospective study. Front Public Health.

[5] Nie LJ, Liang J, Shan F et al. L-Carnitine and Acetyl-L-Carnitine: Potential Novel Biomarkers for Major Depressive Disorder. *Frontiers in Psychiatry*. 2021;12. Accessed March 1, 2023. https://www.frontiersin.org/articles/10.3389/fpsyt.2021.671151. 10.3389/fpsyt.2021.671151 PMC8514700 34658942

[6] Liu Q, He H, Yang J, Feng X, Zhao F, Lyu J (2020). Changes in the global burden of depression from 1990 to 2017: findings from the Global Burden of Disease study. J Psychiatr Res.

[7] Roohi E, Jaafari N, Hashemian F (2021). On inflammatory hypothesis of depression: what is the role of IL-6 in the middle of the chaos?. J Neuroinflamm.

[8] Ting EYC, Yang AC, Tsai SJ (2020). Role of Interleukin-6 in depressive disorder. Int J Mol Sci.

[9] Otte C, Gold SM, Penninx BW (2016). Major depressive disorder. Nat Rev Dis Primers.

[10] Wang J, Wu X, Lai W (2017). Prevalence of depression and depressive symptoms among outpatients: a systematic review and meta-analysis. BMJ Open.

[11] McIntyre RS (2016). Implementing treatment strategies for different types of depression. J Clin Psychiatry.

[12] Hauenstein EJ (2003). Depression in adolescence. J Obstet Gynecol Neonatal Nurs.

[13] Anjum S, Qusar MMAS, Shahriar M, Islam SMA, Bhuiyan MA, Islam MR (2020). Altered serum interleukin-7 and interleukin-10 are associated with drug-free major depressive disorder. Therapeutic Adv Psychopharmacol.

[14] Schneider B, Prvulovic D, Oertel-Knöchel V (2011). Biomarkers for major depression and its delineation from neurodegenerative disorders. Prog Neurobiol.

[15] Das R, Emon MPZ, Shahriar M (2021). Higher levels of serum IL-1β and TNF-α are associated with an increased probability of major depressive disorder. Psychiatry Res.

[16] Suneson K, Lindahl J, Chamli Hårsmar S, Söderberg G, Lindqvist D (2021). Inflammatory Depression—mechanisms and non-pharmacological interventions. Int J Mol Sci.

[17] Li W, Ali T, He K (2021). Ibrutinib alleviates LPS-induced neuroinflammation and synaptic defects in a mouse model of depression. Brain Behav Immun.

[18] Emon MPZ, Das R, Nishuty NL, Shalahuddin Qusar MMA, Bhuiyan MA, Islam MR (2020). Reduced serum BDNF levels are associated with the increased risk for developing MDD: a case-control study with or without antidepressant therapy. BMC Res Notes.

[19] Das R, Emon MPZ, Chowdhury SF, Huque S, Zahan T, Islam MR (2019). Evaluation of serum glial cell line-derived neurotrophic factor in Bangladeshi major depressive disorder patients. Cureus.

[20] Schildkraut JJ (1965). The catecholamine hypothesis of affective disorders: a review of supporting evidence. AJP.

[21] Drevets WC, Frank E, Price JC (1999). PET imaging of serotonin 1A receptor binding in depression. Biol Psychiatry.

[22] Saveanu RV, Nemeroff CB (2012). Etiology of Depression: genetic and environmental factors. Psychiatric Clin.

[23] Kunugi H (2021). Gut microbiota and pathophysiology of depressive disorder. ANM.

[24] Jentsch MC, Van Buel EM, Bosker FJ (2015). Biomarker approaches in major depressive disorder evaluated in the context of current hypotheses. Biomark Med.

[25] Hacimusalar Y, Eşel E (2018). Suggested biomarkers for major depressive disorder. Noro Psikiyatr Ars.

[26] Islam MR, Islam MR, Ahmed I (2018). Elevated serum levels of malondialdehyde and cortisol are associated with major depressive disorder: a case-control study. SAGE Open Med.

[27] Xiu MH, Chen S, Wang F (2008). Altered interleukin-3 serum levels in drug-naïve and neuroleptic-treated Schizophrenic patients. Schizophr Res.

[28] Giralt M, Carrasco J, Penkowa M (2001). Astrocyte-targeted expression of Interleukin-3 and Interferon-α causes region-specific changes in Metallothionein expression in the brain. Exp Neurol.

[29] Dimitrov DH, Lee S, Yantis J (2013). Differential correlations between inflammatory cytokines and psychopathology in veterans with schizophrenia: potential role for IL-17 pathway. Schizophr Res.

[30] Sirota P, Schild K, Elizur A, Djaldetti M, Fishman P (1995). Increased interleukin-1 and interleukin-3 like activity in Schizophrenic patients. Prog Neuro-psychopharmacol Biol Psychiatry.

[31] Farrar W, Vinocour M, Hill J (1989). In situ hybridization histochemistry localization of interleukin-3 mRNA in mouse brain. Blood.

[32] Tabira T, Chui DH, Fan JP, Shirabe T, Konishi Y (1998). Interleukin-3 and Interleukin-3 receptors in the Braina. Ann N Y Acad Sci.

[33] Naudé PJW, den Boer JA, Comijs HC (2014). Sex-specific associations between Neutrophil Gelatinase-Associated Lipocalin (NGAL) and cognitive domains in late-life depression. Psychoneuroendocrinology.

[34] Naudé PJW, Eisel ULM, Comijs HC (2013). Neutrophil gelatinase-associated lipocalin: a novel inflammatory marker associated with late-life depression. J Psychosom Res.

[35] Gouweleeuw L, Naudé PJW, Rots M, DeJongste MJL, Eisel ULM, Schoemaker RG (2015). The role of neutrophil gelatinase associated lipocalin (NGAL) as biological constituent linking depression and Cardiovascular Disease. Brain Behav Immun.

[36] Marijnissen RM, Naudé PJW, Comijs HC, Schoevers RA, Oude Voshaar RC (2014). Waist circumference and Neutrophil Gelatinase-Associated Lipocalin in late-life depression. Brain Behav Immun.

[37] Song C, Wang H (2011). Cytokines mediated inflammation and decreased neurogenesis in animal models of depression. Prog Neuropsychopharmacol Biol Psychiatry.

[38] Nahar Z, Sal-Sabil N, Sohan M, Qusar MS, Islam MR (2023). Higher serum interleukin-12 levels are associated with the pathophysiology of major depressive disorder: a case-control study results. Health Sci Rep.

[39] Smith KM, Renshaw PF, Bilello J (2013). The diagnosis of depression: current and emerging methods. Compr Psychiatr.

[40] Himmerich H, Binder EB, Künzel HE (2006). Successful antidepressant therapy restores the disturbed interplay between TNF-α system and HPA Axis. Biol Psychiatry.

[41] Erta M, Quintana A, Hidalgo J (2012). Interleukin-6, a major cytokine in the Central Nervous System. Int J Biol Sci.

[42] Myint AM, Kim YK, Verkerk R, Scharpé S, Steinbusch H, Leonard B (2007). Kynurenine pathway in major depression: evidence of impaired neuroprotection. J Affect Disord.

[43] Tsao CW, Lin YS, Chen CC, Bai CH, Wu SR (2006). Cytokines and serotonin transporter in patients with major depression. Prog Neuropsychopharmacol Biol Psychiatry.

[44] Audet MC, Anisman H. Interplay between pro-inflammatory cytokines and growth factors in depressive illnesses. *Frontiers in Cellular Neuroscience*. 2013;7. Accessed March 1, 2023. https://www.frontiersin.org/articles/10.3389/fncel.2013.00068. 10.3389/fncel.2013.00068 PMC3650474 23675319

[45] Anisman H, Merali Z, Hayley S (2008). Neurotransmitter, peptide and cytokine processes in relation to depressive disorder: comorbidity between depression and neurodegenerative disorders. Prog Neurobiol.

[46] Islam S, Islam T, Nahar Z (2022). Altered serum adiponectin and interleukin-8 levels are associated in the pathophysiology of major depressive disorder: a case-control study. PLoS ONE.

[47] Riya S, Sultana S, Daria S, et al. Evaluation of serum lysophosphatidic acid and lysophosphatidylcholine levels in major depressive disorder patients. Cureus. 2020;12(12). 10.7759/cureus.12388. 10.7759/cureus.12388 PMC7849208 33542861

[48] Goldsmith DR, Rapaport MH, Miller BJ (2016). A meta-analysis of blood cytokine network alterations in psychiatric patients: comparisons between schizophrenia, bipolar disorder and depression. Mol Psychiatry.

[49] Wohleb ES, Franklin T, Iwata M, Duman RS (2016). Integrating neuroimmune systems in the neurobiology of depression. Nat Rev Neurosci.

[50] Osimo EF, Pillinger T, Rodriguez IM, Khandaker GM, Pariante CM, Howes OD (2020). Inflammatory markers in depression: a meta-analysis of mean differences and variability in 5,166 patients and 5,083 controls. Brain Behav Immun.

[51] Xiu MH, Lin CG, Tian L (2015). Increased IL-3 serum levels in chronic patients with schizophrenia: Associated with psychopathology. Psychiatry Res.

[52] Hercus TR, Barry EF, Dottore M (2013). High yield production of a Soluble Human Interleukin-3 variant from E. Coli with Wild-Type Bioactivity and Improved Radiolabeling Properties. PLoS ONE.

[53] Han QQ, Yu J (2014). Inflammation: a mechanism of depression?. Neurosci Bull.

[54] Ng A, Tam WW, Zhang MW (2018). IL-1β, IL-6, TNF- α and CRP in Elderly patients with Depression or Alzheimer’s Disease: systematic review and Meta-analysis. Sci Rep.

[55] Sapolsky R, Rivier C, Yamamoto G, Plotsky P, Vale W (1987). Interleukin-1 stimulates the secretion of hypothalamic corticotropin-releasing factor. Science.

[56] Mota R, Gazal M, Acosta BA (2013). Interleukin-1β is associated with depressive episode in major depression but not in bipolar disorder. J Psychiatr Res.

[57] Miklowitz DJ, Portnoff LC, Armstrong CC (2016). Inflammatory cytokines and nuclear factor-kappa B activation in adolescents with bipolar and major depressive disorders. Psychiatry Res.

[58] Zou W, Feng R, Yang Y (2018). Changes in the serum levels of inflammatory cytokines in antidepressant drug-naïve patients with major depression. PLoS ONE.

[59] Tuglu C, Kara SH, Caliyurt O, Vardar E, Abay E (2003). Increased serum Tumor necrosis factor-alpha levels and treatment response in major depressive disorder. Psychopharmacology.

[60] Nishuty NL, Khandoker MMH, Karmoker JR, et al. Evaluation of serum Interleukin-6 and C-reactive protein levels in Drug-naïve major depressive disorder patients. Cureus. 2019;11(1). 10.7759/cureus.3868. 10.7759/cureus.3868 PMC6414189 30899619

[61] Al-Hakeim HK, Al-Rammahi DA, Al-Dujaili AH (2015). IL-6, IL-18, sIL-2R, and TNFα proinflammatory markers in depression and schizophrenia patients who are free of overt inflammation. J Affect Disord.

[62] Elgellaie A, Thomas SJ, Kaelle J, Bartschi J, Larkin T (2023). Pro-inflammatory cytokines IL-1α, IL-6 and TNF-α in major depressive disorder: sex-specific associations with psychological symptoms. Eur J Neurosci.

[63] Shashidharamurthy R, Machiah D, Aitken JD (2013). Differential Role of Lipocalin 2 during Immune complex–mediated Acute and chronic inflammation in mice. Arthr Rhuem.

[64] De la Chesnaye E, Manuel-Apolinar L, Oviedo-de Anda N, Revilla-Monsalve MC, Islas-Andrade S (2016). [Gender differences in lipocalin 2 plasmatic levels are correlated with age and the triglyceride/high-density lipoprotein ratio in healthy individuals]. Gac Med Mex.

[65] Jaberi SA, Cohen A, D’Souza C (2021). Lipocalin-2: structure, function, distribution and role in metabolic disorders. Biomed Pharmacother.

[66] Zhang J, Wu Y, Zhang Y, Leroith D, Bernlohr DA, Chen X (2008). The role of lipocalin 2 in the regulation of inflammation in adipocytes and macrophages. Mol Endocrinol.

[67] Ferreira A, Pinto V, Mesquita S et al. Lipocalin-2 is involved in emotional behaviors and cognitive function. *Frontiers in Cellular Neuroscience*. 2013;7. Accessed August 4, 2023. https://www.frontiersin.org/articles/10.3389/fncel.2013.00122. 10.3389/fncel.2013.00122 PMC3725407 23908604

[68] Kang SS, Ren Y, Liu CC (2018). Lipocalin-2 protects the brain during inflammatory conditions. Mol Psychiatry.

[69] Dantzer R, O’Connor JC, Lawson MA, Kelley KW (2011). Inflammation-associated depression: from serotonin to kynurenine. Psychoneuroendocrinology.

[70] Reichenberg A, Yirmiya R, Schuld A (2001). Cytokine-associated emotional and cognitive disturbances in humans. Arch Gen Psychiatry.

[71] Noto C, Ota VK, Gouvea ES (2015). Effects of Risperidone on Cytokine Profile in Drug-Naïve First-Episode Psychosis. Int J Neuropsychopharmacol.

[72] Dahl J, Ormstad H, Aass HCD (2014). The plasma levels of various cytokines are increased during ongoing depression and are reduced to normal levels after recovery. Psychoneuroendocrinology.

